# Borosilicate bioactive glasses with added Mg/Sr enhances human adipose-derived stem cells osteogenic commitment and angiogenic properties

**DOI:** 10.1007/s10856-024-06830-x

**Published:** 2024-11-30

**Authors:** Jenna M. Tainio, Sari Vanhatupa, Susanna Miettinen, Jonathan Massera

**Affiliations:** 1 Bioceramics, Bioglasses and Biocomposites Group, Faculty of Medicine and Health Technology, Tampere University, Tampere, 33720 Finland; 2 Adult Stem Cell Group, Faculty of Medicine and Health Technology, Tampere University, Tampere, 33520 Finland; 3 Tays Research Services, Wellbeing Services County of Pirkanmaa, Tampere University Hospital, Elämänaukio, Kuntokatu 2, 33520 Tampere, Finland

## Abstract

Bioactive glasses are one of the most promising materials for applications in bone tissue engineering. In this study, the focus was on borosilicate bioactive glasses with composition 47.12 SiO_2_ - 6.73 B_2_O_3_ - 21.77-x-y CaO - 22.65 Na_2_O - 1.72 P_2_O_5_ - x MgO - y SrO (mol%). These compositions are based on silicate S53P4 bioactive glass, from where 12.5% of SiO_2_ is replaced with B_2_O_3_, and additionally, part of CaO is substituted for MgO and/or SrO. The impact of ion release, both as extract and in direct contact, on human adipose-derived stem cells’ (hADSCs) viability, proliferation, ECM maturation, osteogenic commitment and endothelial marker expression was assessed. Osteogenic media supplements were utilized with the extracts, and in part of the direct cell/material culturing conditions. While it has been reported in other studies that boron release can induce cytotoxicity, the glasses in this study supported cells viability and proliferation. Moreover, borosilicate’s, especially with further Mg/Sr substitutions, upregulated several osteogenic markers (such as *RUNX2a, OSTERIX, DLX5, OSTEOPONTIN*), as well as angiogenic factors (e.g., *vWF* and *PECAM-1*). Furthermore, the studied glasses supported collagen-I production even in the absence of osteogenic supplements, when hADSCs were cultured in contact with the glasses, suggesting that while the bioactive glass degradation products are beneficial for osteogenesis, the glasses surface physico-chemical properties play a significant role on hADSCs differentiation. This study brings critical information on the impact of bioactive glass compositional modification to control glass dissolution and the subsequent influence on stem cells proliferation and differentiation. Furthermore, the role of the material surface chemistry on promoting cell differentiation is reported.

**Graphical Abstract:**

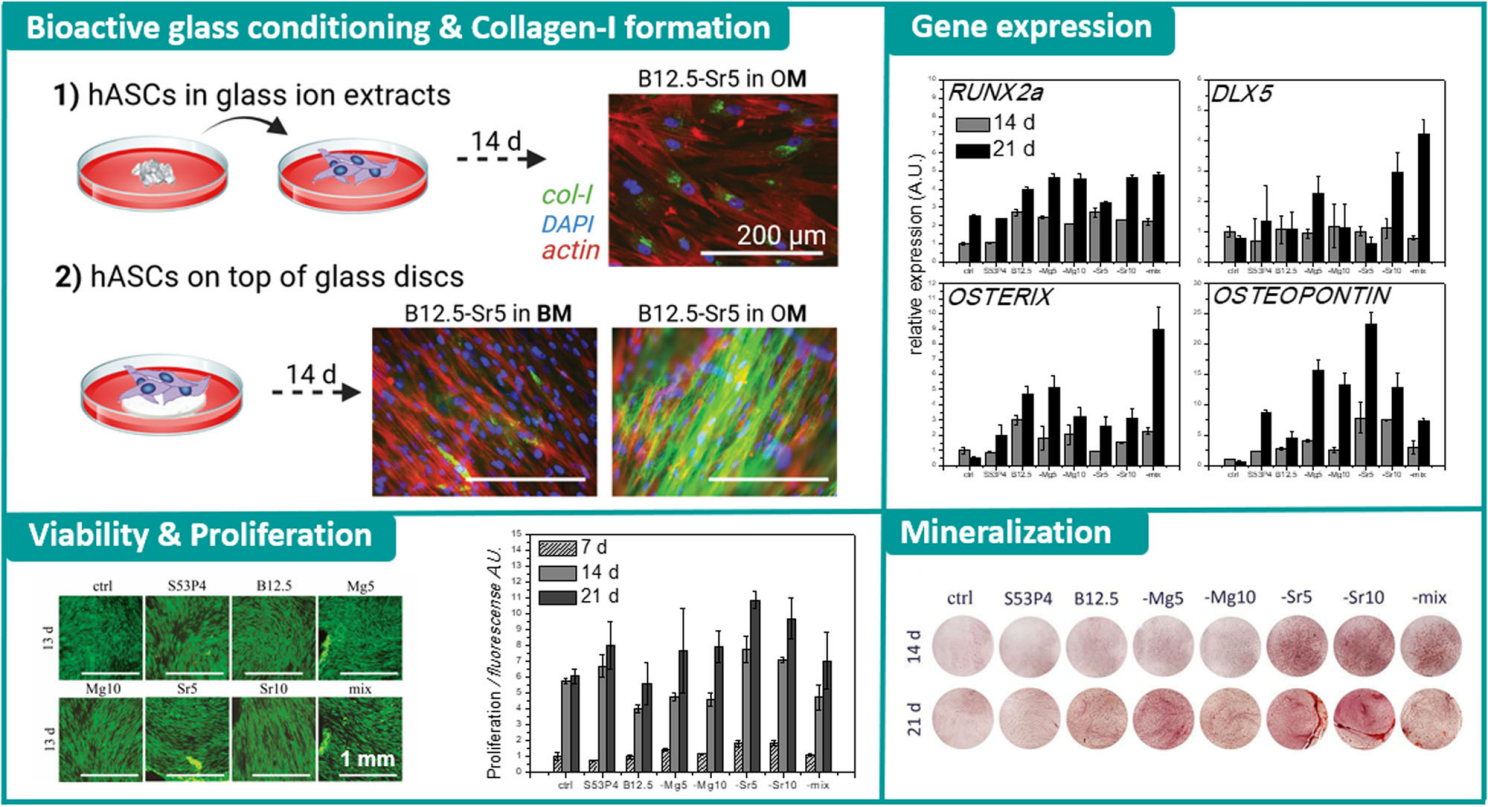

## Introduction

Bone is one of the most transplanted tissues after blood, with ever increasing need for patients missing hard tissue due to large fractures, tumour removal or trauma. One of the aims of evolving fields of regenerative medicine and tissue engineering is to develop more available synthetic alternatives to compensate the need of transplants and tissue grafts. An interesting and promising materials for reconstruction of bone defects are bioactive glasses [1–4]. Discovered already in 1969 by Larry Hench, bioactive glasses are material group that are able to form active bonding with bone tissue. Over the years, further modifications and different composition have been studied to enhance the suitability for support of new bone formation [5–7]. One of the subcategories of bioactive glasses are borosilicate glasses, that offer great potential for both soft and hard tissue engineering applications [8, 9].

Borosilicate bioactive glasses have many benefits when compared to more traditional, commercial silicate compositions; including increasing conversion into hydroxyapatite [10] and additionally enhanced thermal properties enabling more complex processing by heat treatment [11, 12]. However, the development of new bioactive glasses is interdisciplinary; in addition to compositions effect on material properties, it is important to understand the impact of bioactive glasses degradation products on cellular processes. For example, it has been studied that calcium [13], phosphorus [14], silicon [15, 16], boron [9, 17], magnesium [18] and strontium [19, 20] are known to partake in bone metabolism and new bone formation [21].

Borate-based bioactive glasses have been viewed promising to stimulate new bone formation, yet, role of boron on osteogenesis/angiogenesis, as well as the half maximal inhibitory concentration are not yet fully understood [9, 17, 22]. One concern with borate and borosilicate bioactive glasses lies in their potential toxic nature, in vitro, due to rapid release of boron in large concentration [23, 24]. Furthermore, the rapid dissolution of borate glasses, as well as borosilicate glasses with large boron content, leads to significant rise in pH. However, it was shown that pH within the 7.9–8.27 range, while not interfering with human bone marrow-derived mesenchymal stem cells proliferation, inhibited osteogenic proliferation [25]. The potential cytotoxic effect of the boron release can be alleviated by more dynamic testing protocols [23], however, control over ion release remain critical to the success of the bone graft.

Control of the glass dissolution can be achieved through modification of the glass composition. Mg and Sr are widely studied ions, as substitute for Ca [12, 25, 26]. Mg and Sr were both found to reduce the speed of dissolution of silicate and borosilicate bioactive glasses [12]. Indeed, Mg and Sr were both found to stabilize the borate sub-network [12]. Furthermore, Mg and Sr reduce the speed of hydroxyapatite formation aside from being incorporated within the reactive hydroxyapatite layer [12]. Strontium has been found to modulate the osteogenic activity of human bone marrow-derived mesenchymal stem cells [27]. Strontium has also been found to increase osteoblast proliferation and ALP activity while inhibiting TRAP activity [19]. Magnesium-containing bioactive glasses have been widely studied in vitro and in vivo. Mg doped bioactive glasses have been found to cause no cytotoxicity towards osteoblastic cells, enhance bone nodule formation and promote migration of human endothelial cells [28].

It should be noted that, generally, in vitro testing of bioactive glasses has been difficult to compare between studies due to the significant variation in the protocol used (cell lines, cell types, sample shape/size, pre-incubation etc.). Furthermore, while bioactive glass can be used alone, many studies report the impact of bioactive glass ion release through polymeric matrices (composite, hybrid, cements). Finally, while ion release is expected during cell culture, the exact ionic concentration in the cell culture medium, at each time point is rarely reported. It is therefore of paramount importance to not only study the impact of ion release (ion concentration) on cells behaviour but also to assess the role of the materials surface/cell interaction on the cell fate. Here, hADSCs have been used. Easily available hADSCs are frequently used in research, and additionally successfully in cell therapies, due to their bone differentiation capacity in vitro and bone regeneration capacity in vivo [29, 30]. The compositions of the borosilicate glasses in this study are based on well-known, commercialized bioactive glass composition S53P4 (BonAlive ®) [2, 31]. B12.5-glass series’ in vitro reactivity has been studied simulated body (SBF) fluid has been previously studied [12].

In this study, the aim was to investigate the effect of the B12,5 glass series’ degradation products on hADSC in vitro. The impacts on viability and proliferation were observed, as well as the dissolution products osteostimulative properties were evaluated by assessing the differentiation towards osteoblastic cell lines. Osteogenesis-inducing chemicals of β- glycerophosphate, L-ascorbic acid 2-phosphate and dexamethasone were supplemented with the glass extracts [32]. Additionally, the stimulation of the expression of few endothelial markers was analysed, as borosilicate bioactive glasses are known have to support angiogenesis [8, 9]. Moreover, the viability and differentiation of the hADSCs were additionally assessed in direct contact with the material by culturing hADSC on top of glass discs.

## Materials and methods

### Bioactive glass preparation and pre-treatment

The studied borosilicate glasses 47.12 SiO_2_ – 6.73 B_2_O_3_ – 21.77 (-x-y) CaO – 22.65 Na_2_O – 1.72 P_2_O_5_ – x MgO – y SrO (mol-%), where x,y = 0, 5 or 10 mol-% are presented in Table 1. The base borosilicate glass composition (x,y = 0), has been referred as B12.5 [12]. Batches of glass were melted in a platinum crucible from mixtures of sand (99,4% of pure SiO_2_) and analytical grade reagents from Sigma-Aldrich (H_3_BO_3_, MgO, SrCO_3_, (NH_4_)H_2_PO_4_, Na_2_CO_3_) and ThermoFisher (CaCO_3_) in an electric furnace (P310, Nabertherm GmbH) under air atmosphere at 1300 °C. Melts were casted into pre-heated cylindrical (d = 10 mm) graphite mold to obtain glass rods, and annealed at 40 °C below the glasses’ respective glass transition temperatures. The obtained rods were either cut into discs (h = 2 mm), that were polished up to 4500 grit, or grounded into particles (fraction size 500–1000 µm). Prior to use in cell culture experiments, processed discs and glass particles were heated at 200 °C for 1 h, and later disinfected in 70% ethanol (2 × 10 min at RT) in the cell culture laminar hood.

**Table 1:** Oxide compositions of the studied glasses in mol-%

*Glass composition*		*SiO*_*2*_	*B*_*2*_*O*_*3*_	*CaO*	*Na*_*2*_*O*	*P*_*2*_*O*_*5*_	*MgO*	*SrO*
	S53P4	53.85	–	21.77	22.66	1.72	–	–
	B12.5	47.12	6.73	21.77	22.66	1.72	–	–
‘Mg5’	B12.5-Mg5	47.12	6.73	16.77	22.66	1.72	5	–
‘Mg10’	B12.5-Mg10	47.12	6.73	11.77	22.66	1.72	10	–
‘Sr5’	B12.5-Sr5	47.12	6.73	16.77	22.66	1.72	–	5
‘Sr10’	B12.5-Sr10	47.12	6.73	11.77	22.66	1.72	–	10
‘mix’	B12.5-Mg5-Sr10	47.12	6.73	6.77	22.66	1.72	5	10

### Cell culture and media

This study was conducted in accordance with the ethical approval granted by the Ethics Committee of Pirkanmaa Hospital District, Tampere, Finland (R15161). The hADSCs were isolated from abdominal subcutaneous adipose tissue sample collected at the Tampere University Hospital Department of Plastic and Reconstructive Surgery from a 62-year-old male donor in 2015. Isolation of the hADSCs and characterization of the surface marker profile as well as adipogenic and osteogenic differentiation potential were performed as described previously [33, 34]. Cells were able to differentiate toward adipogenic and osteogenic lineages. At passage 1, the cells highly expressed CD105 (96%), CD73 (81%) and CD90 (99%), and had low expression of CD45 (4%), CD34 (4%), CD14 (0,3%), CD19 (0,2%) and HLA-DR (0,4%) cell surface molecules. Experiments related to bioactive glass were performed with hADSC in passages 5–7. Basic growth media (BM) consisted of α-Minimum Essential Media (α-MEM; Gibco, Life Technologies), with 5% human serum (BioWest) and 1% penicillin/streptomycin (Lonza, Biowhittaker), providing an animal origin free culture conditions that represents the natural growth environment of human-originated cell. The BM conditions were utilized with cell cultures grown on top of the glass discs. Additionally, as a comparison, cultures were grown in the presence of osteogenic media (OM) supplements; with addition of 10 mM β- glycerophosphate, 250 µm L-ascorbic acid 2-phosphate and 1 µl/ml dexamethasone (Sigma-Aldrich) to BM.

Glass ion-conditioned extracts were prepared from the granules similarly as described in by Ojansivu et al. [32]. Briefly, 2.1 g glass particles of fraction size 500–1000 µm per 24 ml of α-MEM, were incubated 24 h in a cell culture petri dish of 10 cm diameter (Corning). After incubation, the media containing dissolution products were collected and sterile filtered, before 5% addition of serum, 1% penicillin-streptomycin and OM supplements. New extract batches were prepared weekly, and OM supplements were freshly added before exchange of media.

Cells were cultured at 37 °C in a humidified atmosphere of 5% CO_2_ balanced 95% air in incubator (Thermo Scientific forma steri-cycle i160 CO^2^). Cells were plated in BM and on the following day (day 0 of experiments), proper media was introduced (ion extract, or for the disc cultures either BM or OM). As an addition to the bioactive glass conditions, control cultures were grown without bioactive glass products. For ion extract cultures, controls were grown only in presence of pure OM. For cultures grown directly on top of discs, controls cells were cultured on polysterene (cell culture well plate), with the presence of the BM/OM. The media (300 µl per 48-wellplate well) was changed every 3-4 days, up to 21 culturing days. For glass ion extract studies, hADSCs were seeded at a density of ~160 cells per cm^2^ in 48-well plates (Corning). For culturing on top of the bulk glass discs, the discs were immersed overnight in the BM upon plating of the cells, to reduce the effect of initial burst of ions upon from the bioactive glass [35]. Cell seeding density of the direct contact cultures was for viability testing 3200 cells per cm^2^, and for immunocytochemical studies 2200 cells per cm^2^.

The culture media and glass dissolution extracts were first stored at −20 °C, and later thawed and analysed for ion concentration by inductively coupled plasma optical emission spectrometry (ICP-OES, Agilent Technologies). Possible precipitates were dissolved using ultrasonic bath. The samples were diluted 1:10 in distilled water and spiked with 50 µl of 70% ultrapure HNO_3_ per 10 ml sample before the analysis. Analysed elements were Si (at wavelength 251.611 nm), B (249.772 nm), Ca (317.933 nm), Na (589.592 nm), P (213.618 nm), Mg (285.213 nm) and Sr (216.596 nm).

### Assessment of cell viability and proliferation

Cell viability was evaluated both with ion extracts, and on direct culturing on top of glass discs in OM and BM with the hADSCs. Viability was analysed with Live/Dead staining (Invitrogen, Thermo Fisher Scientific) up to 21 days of culturing as previously described [24]. Briefly, the cultured cells were incubated in 0.25 µM Ethidium homodimer-1 (EthD-1) and 0.5 µM Calcein-AM containing solution for 20 min at room temperature (RT), followed by immediate imaging (IX51 inverted microscopy, Olympus, equipped with a fluorescence unit and a camera DP30BW, Olympus). EthD-1 was imaged at 546 nm and Calcein-AM at 488 nm.

Cell proliferation was evaluated for ion extracts cultures based on total DNA amount after 7, 14 and 21 days of culture with CyQUANT Cell Proliferation Assay (Invitrogen, Thermo Fisher Scientific), according to the manufacturer’s protocol. Briefly, cells were lysed in 0.1% TritonX-100 lysis buffer (Sigma-Aldrich), and the collected supernatant stored −80 °C until the analysis. After the freeze-thaw cycle, 20 µl of three parallel replicates of each lysate were pipetted to a 96-well plate (Nunc) and mixed with 180 µl working solution (CyQUANT GR dye and lysis buffer 1:1). The fluorescence was measured with a multiple plate reader (Victor 1420 Multilabel counter, Wallac) at 480/520 nm.

### Alkaline phosphatase activity and gene expression

Quantitative alkaline phosphatase (qALP) activity, and the relative expression of osteogenic and angiogenic marker genes by quantitative real-time reverse transcription polymerase chain reaction (qRT-PCR) were assessed only for ion extract cultures.

Alkaline phosphatase activity was studied quantitatively as described previously [32]. Briefly, 20 µl of cell lysate (taken from same sample as for the proliferation assay) was combined with 90 µl working solution (1:1 mixture of alkaline buffer solution and phosphatase substrate, Sigma-Aldrich) in a 96-well plate. After 15 min incubation at +37 °C, the reaction was stopped by adding 50 µl 1 M NaOH (Sigma-Aldrich). Absorbance was measured with Victor 1420 Multilabel counter (Wallac) at 405 nm.

For qRT-PCR gene expression study, cells were cultured for 14 and 21 days. RNA isolation was performed using Macherey-Nagel NucleoSpin-kit according to the manufacturer’s protocol. RNA concentrations were measured with a spectrophotometer (Nanodrop 2000; Thermo Fisher) and stored at −80 °C prior cDNA transcription (25 ng/µl) with High-Capacity cDNA Reverse Transcriptase Kit (AppliedBiosystems, Life Technologies). The studied genes included human osteogenic markers; runt-related transcription factor 2a (*RUNX2a*), transcription factor Sp7; *OSTERIX*, homeobox protein *DLX5*, secreted phosphoprotein 1; *OSTEOPONTIN*, and *OSTEOCALCIN*, as well as of endothelial markers; von Willebrand factor (*vWF*) and platelet endothelial cell adhesion molecule-1 (*PECAM-1*). The expression of housekeeping gene human acidic ribosomal phosphoprotein P0 (*RPLP0*) was used to normalize the data of expression levels between samples. Primers were from Oligomer (*RPLP0*, *RUNX2a*, *OSTERIX, DLX5, OSTEOPONTIN*), DNA technologies (*OSTEOCALCIN*), and Qiagen (*vWF, PECAM-1*). The primer sequences and the mRNAs accession numbers were as previously presented [24]. qRT-PCR was performed with QuantStudio 12 K Flex Real-Time PCR System (Applied Biosystems) and the relative expressions were calculated with a mathematical model by Pfaffl [36].

### Immunocytochemical staining and mineralization assay

Immunocytochemical staining for collagen-I were performed on both extracts and disc conditions, osteocalcin only for the disc cultures. The mineralization assay (Alizarin red) was conducted only for the extract cultures.

Immunocytochemical staining was performed after 7, 14 and 21 days of culture as previously described [24]. Briefly, cultured cells were fixed with 4% paraformaldehyde (PFA) for 30 min at RT, and nonspecific staining was blocked by incubation with 1% bovine serum albumin (BSA) for 60 min at +4 °C. Primary antibody against collagen I (mouse monoclonal anti-collagen-I, 1:2000, Abcam) and against osteocalcin (mouse monoclonal anti-osteocalcin 1:100, Abcam) were incubated on cell samples overnight (+4 °C.) After following phosphate buffered saline (PBS) washes, cultures were incubated with secondary antibodies (donkey anti-mouse Alexa fluor 488 IgG, 1:800 Invitrogen, Thermo Fisher Scientific) together with actin-staining phalloidin-TRITC (1:500, Sigma-Aldrich). Additionally, after the washing steps after the secondary antibody treatment, the samples were incubated with PBS including 4′,6-diamidino-2-phenylindole (DAPI; 1:200, Molecular Probes, Thermo Fisher Scientific) to stain the nuclei. Cultures were imaged with microscope with fluorescent unit (IX51, Olympus).

Alizarin red staining was utilized to evidence calcium phosphate (CaP) mineral formation. The assay was performed after 14 and 21 days of culture. In brief, cells were fixed with iced cold 70% EtOH (incubation 90 min at −20 °C) and stained with 2% Alizarin red S (pH 4.1–4.3; Sigma-Aldrich) solution for 10 min at RT. After PBS washes, the samples were imaged. For quantified measurements, the attached dye was extracted with 100 mM cetylpyridinium chloride (Sigma-Aldrich) and absorbances were measured at 544 nm (Victor 1420 Multilabel counter).

## Results

### Glass degradation products in cell culture media

ICP-OES analysis was performed on the extracts to determine the concentration of the released ions in the media, and these results are presented for Si, B, Mg, Sr, Ca and P in Table 2. Na, one of the elements present in the glass as well as in the original medium, was analysed but exact values not reported due to oversaturation (exceeding calibration limit of 400 ppm). As seen, the culture medium, and thus also the control conditions, did not contain Si, B or Sr, that were, therefore, introduced by the glass degradation.

**Table 2:** Ion concentration (ppm – mg/l) of the culture media base, and extracts after 24 h dissolution of the [500,1000] µm glass particles

*Glass/condition*	*Si*	*B*	*Ca*	*P*	*Mg*	*Sr*
Culture media base	–	–	62 ± 3	31 ± 2	14 ± 1	–
S53P4	54 ± 3	–	176 ± 9	22 ± 1	13 ± 1	–
B12.5	57 ± 3	36 ± 2	167 ± 8	13 ± 1	13 ± 1	–
B12.5-Mg5	56 ± 3	32 ± 2	140 ± 7	25 ± 1	34 ± 2	–
B12.5-Mg10	56 ± 3	33 ± 2	114 ± 6	29 ± 1	57 ± 3	–
B12.5-Sr5	54 ± 3	28 ± 1	131 ± 7	22 ± 1	13 ± 1	73 ± 4
B12.5-Sr10	53 ± 3	27 ± 1	101 ± 5	24 ± 1	13 ± 1	141 ± 7
B12.5-Mg5-Sr10	58 ± 3	36 ± 2	82 ± 1	25 ± 1	34 ± 2	178 ± 4

### Viability and proliferation

**Fig. 1:**
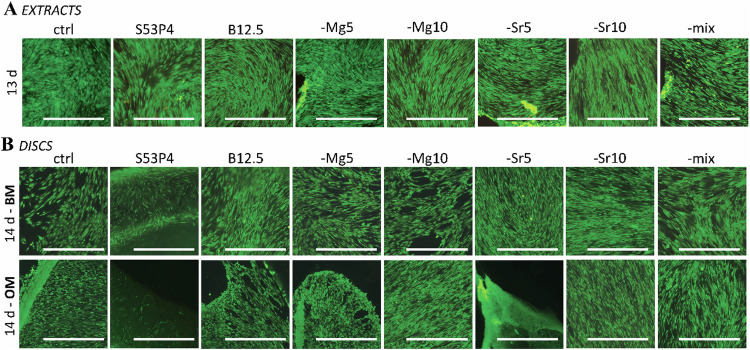
hADSC viability determined via live-dead staining **A** in extracts (with OM supplements) on culturing day 13 and **B** on top of discs; both BM (upper row) and OM (lower row) medias on culturing day 14. Calcein-AM staining (viable cells) presented as green, EthD-1 (dead cells) as red. Scale bar 1 mm for all images

Cell viability was investigated via live/dead staining, and results after 13-14 culturing days are presented in Fig. 1. As can be seen, hADSC were highly viable both in presence of glass dissolution products (as undiluted ion extracts; Fig. 1A) and in direct cell/material contact (on top of glass discs; Fig. 1B).

**Fig. 2:**
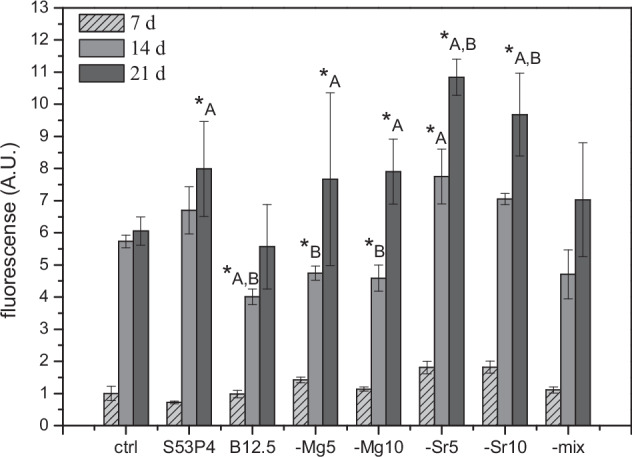
The proliferation of hASDSC measured with CyQuant cell proliferation assay, on control condition and exposed to glass extracts, after 7, 14 and 21 days of culturing. Statistically significant difference (*p* < 0.0021) on the proliferation, when all studied glasses were compared to control condition, is marked with (*_A_), and the statistically significant difference when borosilicate’s were compared with S53P4 extract, is marked with (*_B_)

The proliferation of cells in conditioned media was analysed with CyQuant assay (Fig. 2). Statistical analysis was performed with two-way ANOVA. In all conditions, cell numbers increased gradually through the observation period. After a week of culturing, there was no statistical difference between the conditions. After two weeks, cells proliferation in medium conditioned with B12.5 was lower than in medium conditioned with B12.5Sr5. After three culturing weeks, there was no statistical difference between control, B12.5 and B12.5-Mg5-Sr10 conditions media, while media conditioned with bioactive glass exhibited significantly (*p* ≤ 0.0021) higher proliferation. When comparing the borosilicate’s to the silicate extract, proliferation on S53P4 was significantly higher at 14 and 21 days of culture (*p* ≤ 0.0001), than on pure borosilicate B12.5. On magnesium doped conditioned cultures (glasses labelled “Mg5”, “Mg10” and “mix”) reached similar proliferation as S53P4 extract after 21 days of culturing. On glasses, where calcium was substituted only by strontium (glasses labelled “Sr5” and “Sr10”), the proliferation was not significantly different from S53P4 cultures for up to two weeks, however after 21 days, they exhibited significantly (*p* ≤ 0.0021) higher proliferation.

### Indicators of osteogenic commitment – ALP and gene expression

**Fig. 3:**
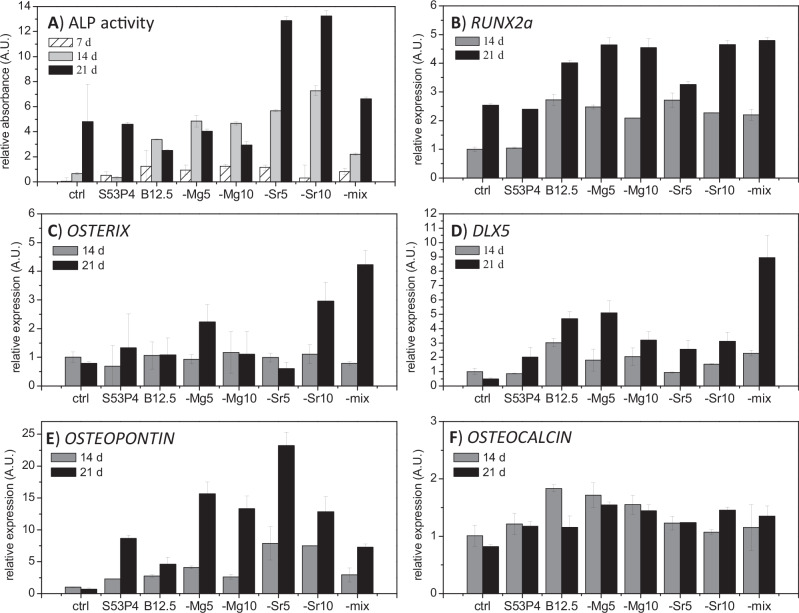
Results for **A** adjusted ALP activity, and **B** *RUNX2a*, **C** *OSTERIX*, **D** *DLX5*, **E** *OSTEOPONTIN* and **F** *OSTEOCALCIN* relative expression in glass ion extract studies up to 21 culturing days

The in vitro osteogenic differentiation of the cells can be divided into three overlapping phases; proliferation, matrix maturation and mineralization. Here, qALP activity was utilized as an indicator of early osteogenic commitment [37]. Some of the most important osteogenic markers (*RUNX2a*, *OSTEOPONTIN, OSTERIX, DLX5*, and *OSTEOCALCIN)* were analysed by their relative expression to additionally assess the osteogenic commitment. Results are presented in Fig. 3, on arbitrary scale, that are adjusted to control levels at the first time point.

Figure 3A presents the results for qALP. Cells cultured on control (osteogenic media without glass ion extracts) and S53P4 conditioned media show an ALP activity that remains low until a major increase is observed after 21 days of culturing. For the borosilicate conditioned cultures, major increase in ALP activity could be seen already after 14 days of culturing. ALP activity seemed to decrease on borosilicate conditions without Sr after 21 culturing days, while, for Sr-containing ion extract cultures, expression was gradually increasing through the 7–21 days of culturing.

*RUNX2a* expression (Fig. 3B) was similar for the control (osteogenic media) and S53P4 conditioned media. *RUNX2*a, an early osteogenic marker, was upregulated for the whole borosilicate series, and gradually increased after both 14 and 21 days of culture. The expression of this gene is essential for osteoblast formation. For the osteogenic differentiation to proceed, the expression should decrease to enable mineralization [38]. *OSTERIX* (Fig. 3C) protein is a bone specific transcription factor, required for osteoblast differentiation and essential for mineralization [39]. At 14 days, the expression of *OSTERIX* was on a similar level in all conditions. After 21 days, variations between the cultures were observed; upregulation was observed for B12.5-Mg5, -Sr10 and mixture glass B12.5-Mg5-Sr10. All studied bioactive glasses enhanced higher expression of homeobox protein *DLX5* (Fig. 3D) than on control conditions after 21 culturing days, as seen previously [24]. *DLX5* is a protein coding gene involved in osteoblast differentiation and induction of mineralization [40]. *OSTEOPONTIN* (Fig. 3E) is a protein coding gene for major non-collagenous bone protein that binds tightly to hydroxyapatite [41]. All bioactive glasses demonstrated a notable higher expression than on control conditions on both studied time points. After 21 days of culturing, expression was slightly downregulated on control condition. Expression of *OSTEOCALCIN* (Fig. 3F) was not as clearly regulated by any of the glass extracts, as some of the other studied genes.

### Endothelial marker expression

**Fig. 4:**
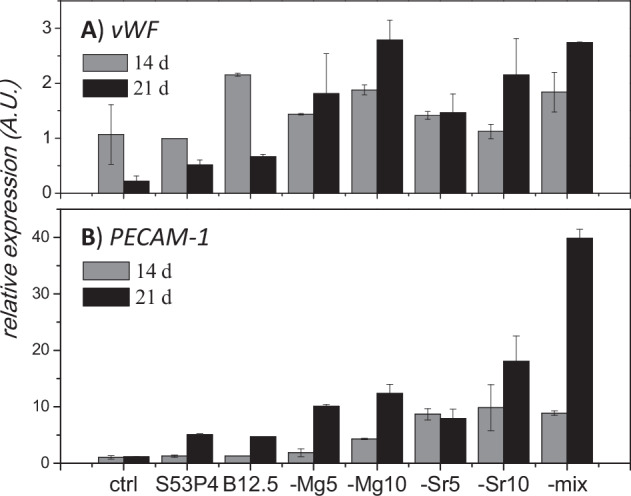
Adjusted relative endothelial marker expression, determined with qRT-PCR; **A** vWF and **B** PECAM-1 expression after 14 and 21 culturing days

As bioactive glasses, especially with boron in their composition, are known to support angiogenesis [9, 17, 21], the degradation products’ effect on expression of endothelial factors *vWF* [42] and *PECAM-1* [43] was assessed. The qPCR results for these gene expressions are presented in Fig. 4. With control, S53P4 and B12.5 cultures, the *vWF* expression decreased between 14 and 21 days of culturing, while expression increased in the Mg/Sr substituted glass extract cultures. Additionally, while expression of *PECAM-1* (Fig. 4B) was upregulated by S53P4 and B12.5 cultures after 21 culturing days, the Mg/Sr substituted compositions had a notably higher upregulating effect on the *PECAM-1* expression, increasing with increasing Mg/Sr for Ca substitution in the glass and extract composition.

### ECM maturation and mineralization

**Fig. 5:**
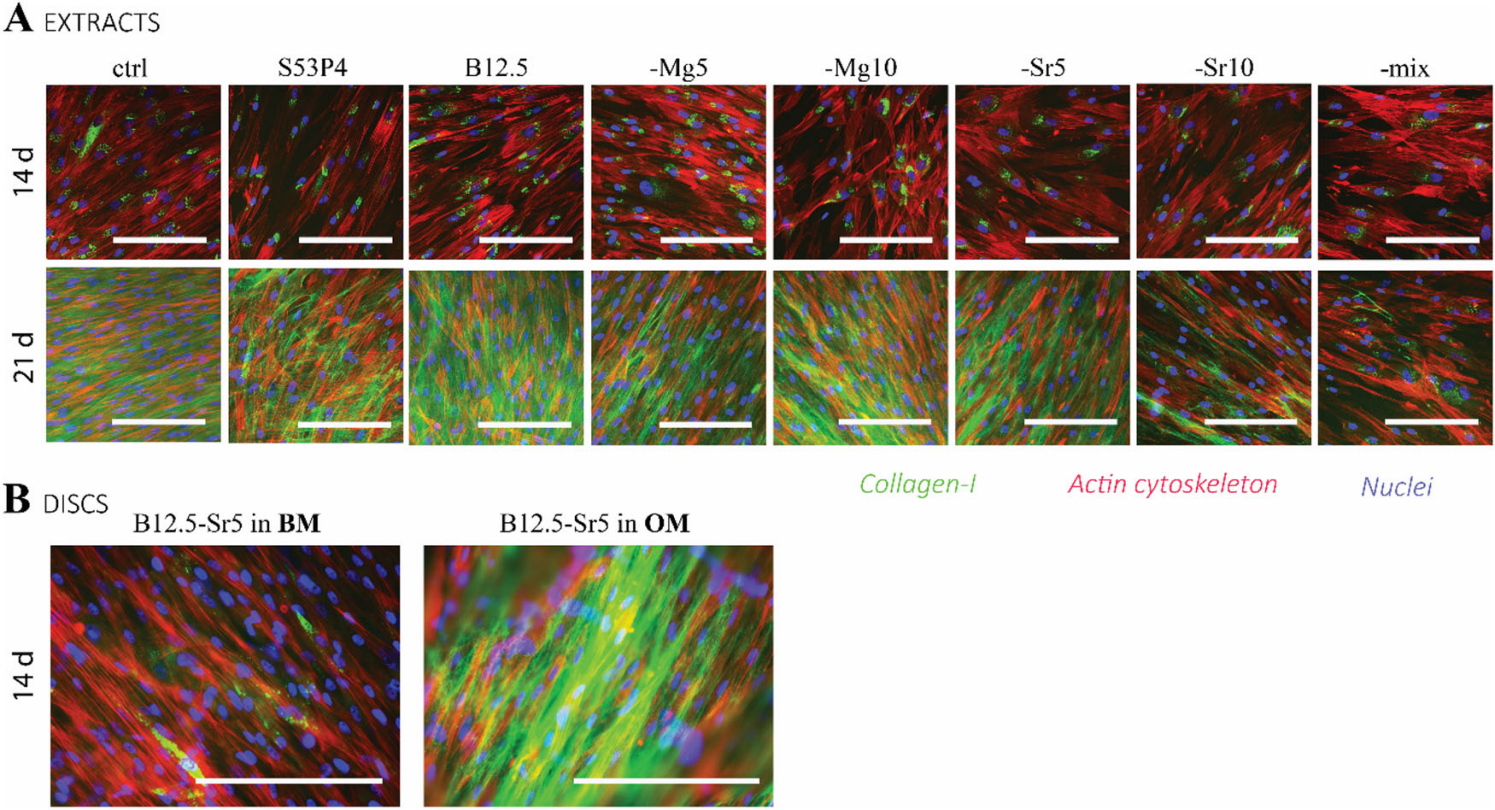
Immunocytochemical staining to observe collagen-I formation in **A** ion extract cultures after 14 and 21 days of culturing, and **B** disc culture examples after 14 days of culturing; B12.5-Sr5 glass both in BM and OM media. Staining’s presented for col-I (green), actin network (red) and cell nuclei (blue). Scale bar 200 µm in all of the images

Bone is a connective tissue characterized by a mineralized ECM, which major structural components consists of type I collagen fiber network and a mineral phase mainly as calcium phosphate, in the form of hydroxyapatite crystals [44]. In this respect, immunocytochemical staining for collagen-I and mineralization assays were utilized to evaluate the osteogenic differentiation as well as the maturation of the ECM. Figure 5 presents results for the collagen-I immunocytochemical staining after 14 and 21 days of culturing in ion extracts (Fig. 5A), and examples of B12.5-Sr5 glass disc cultures in both studied media after 14 days (Fig. 5B).

With ion extract cultures (Fig. 5A), intracellular collagen-I expression could be observed with all conditions after 14 days of culturing, and after 21 days, extensive extracellular matrixes could be detected. The results for osteocalcin staining were quite weak, on similar level as background control staining’s, and therefore not presented.

**Fig. 6:**
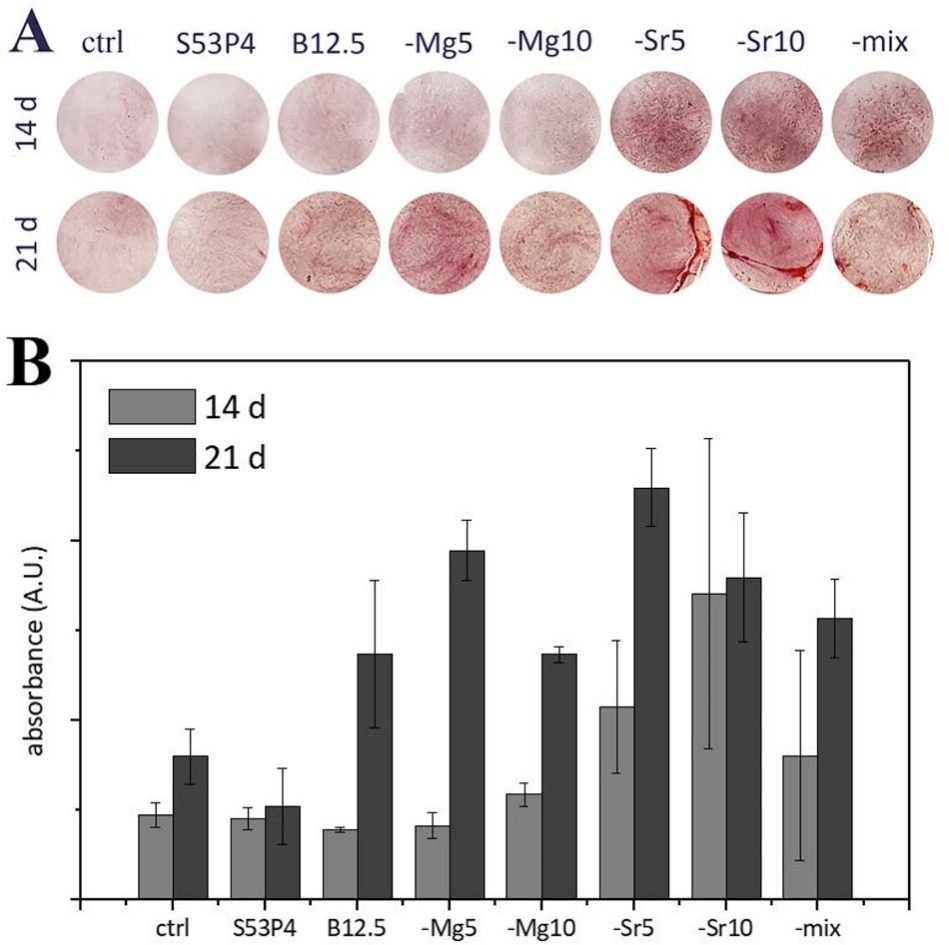
Alizarin red staining for mineralization evaluation. **A** Images of stained cultures after 14 and 21 days, and, **B** Quantified results for the presented Alizarin red staining

To assess the late osteogenesis of hASCs cultured in glass extracts, Alizarin red staining was performed to evidence the mineral formation on cultures, and these results are presented in Fig. 6. Cells cultured on discs were not characterized, as the mineral formed by the cells cannot be discriminated from the HA formed due to the glass reactivity. After the imaging of the staining (Fig. 6A), the stain was extracted and quantitively analysed (Fig. 6B).

## Discussion

When the glasses reactivity was studied in SBF [12], it was seen that while Mg and/or Sr substitution resulted in a slower ion release, the release of Mg/Sr was correlated to the boron release. This behaviour was also observed in the cell culture media extracts. However, within the limits of accuracy (±4 ppm), the level of Si (53–58 ppm) and B (27–36 ppm) in the culture media were similar in the studied borosilicate glasses, irrespective of the glass composition. While an increase of Ca was expected upon glass dissolution, the decrease in the P levels indicates precipitation of a reactive hydroxyapatite (HA) layer, due to early reaction of the bioactive glass during the extract preparation. Glasses containing Mg and/or Sr show higher P levels (22–29 ppm), when compared to B12.5 composition (~13 ppm). Such delay in the HA precipitation has already been discussed extensively in previous studies, and due to the faster dissolution/reaction of the B12.5 glass [12, 45, 46]. Overall, the released ions were otherwise well in line with previous studies for all glass compositions [12, 22, 32].

The cell cultures became highly confluent already after 7 days, and as also seen from cultures on top of the discs (especially OM specimen, Fig. 1B), started to form thick cell matts which partly detached already around the two-week time point. In a previous study with similar type of bioactive glasses [32], it was found that hADSC morphology was affected by borosilicate glasses; enlarged morphology and partial actin network disruption was evidenced, when cells were grown on glass discs. Indeed, also in this study, occasionally some actin network disruption (normal actin cytoskeleton network organisation / function compromised) was observed with hADSCs, that were cultured in contact with the borosilicate glass discs.

In static in vitro cultures, boron has been observed to be toxic to cells when exceeding quite small concentrations [47]. This effect is usually diminished in dynamic culturing conditions and in vivo [8, 48]. In study by Ojansivu et al. [24]. S53P4-based borosilicate glasses B50 and B25 were found to decrease the proliferation of hADSC cultures, as already reported in other studies on borate and borosilicate bioactive glasses [23]. However, in the current study, cultures with B12.5 variants were thriving; the difference could be explained by lower boron levels, than on B50 and B25, where 50 and 25%, respectively, of the SiO_2_ was substituted for B_2_O_3_. In B12.5, only 12.5% of the SiO_2_ was substituted. Despite the lower B_2_O_3_ content in the B12.5, such results were perhaps unexpected, as borate and borosilicate glass’s ability to support cell viability has been generally poorer than those of the corresponding silicate glasses [23, 24, 49]. Based on these results, it could be assumed that the boron concentration in the studied conditions lands in the therapeutic window of the used hADSCs, while both magnesium and strontium substitutions improved the cells proliferation in boron containing environment. The improved cell proliferation can either be due to the beneficial impact of those ions on cells behaviour and/or to the decrease in ion release rate. Indeed, as shown in [12] Mg and/or Sr helps in controlling the boron release owing to the stabilization of the borate network.

At the studied timepoints, increasing *RUNX2a* expression was evidenced in all conditions. Additionally, *DLX5* upregulation by all bioactive glass extracts indicates osteoblast differentiation and induction of mineralization. *OSTERIX* was clearly stimulated only by B12.5-Mg5, -Sr10 and B12.5-Mg5-Sr10. For *OSTEOPONTIN*, the upregulation in the presence of bioactive glass ions was even over 20-fold when compared to the control conditions; as the expression was highly stimulated, it could be attributable to peaking during mineralization phase. However, *OSTEOCALCIN*, expressed by mature osteoblasts was not clearly regulated by any of the glass extracts. It should be kept in mind that the mesenchymal stem cells differentiation towards osteogenic lineages were supported by supplementing the media with L-ascorbic acid 2-phosphate, dexamethasone and beta-glycerophosphate [34]. Overall, results suggested that the bioactive glass dissolution products supported osteoblast differentiation, moving into mineralisation phase. From the endothelial marker expression it seems that the dissolution products could additionally support the angiogenesis of the surrounding tissue. In study by Ojansivu et al. [24], the borosilicate bioactive glasses B25 and B50 had an upregulating effect on these markers, and indeed, B12.5-series seems to have a similar effect. Furthermore, the presence of Mg has also been linked to potential angiogenesis as in ref. [28].

As collagen-I is produced during the osteogenic commitment of stem and progenitor cells [41], the observed fibril-like extracellular collagen-I could be taken as an indication of cells differentiation and maturation. Similarly, in contact with bioactive glasses, hADSCs secreted collagen on the surface of the reactive glass [50] (Fig. 5B). After 14 days of culturing, the BM cultures expressed intracellular collagen-I formation, while on OM cultures the collagen-I was already secreted into the extracellular space. However, when comparing the effect of direct contact culturing to ion extract conditions, it could be observed that after 14 days of culturing, the collagen-I was in similar stage in BM media cultures as seen in OM supplemented ion extracts, while the OM supplemented disc cultures showed even more enhanced collagen production and ECM secretion. It indeed has been seen in previous studies [22, 32] that the role of direct cell-bioactive glass interactions could have more significant effect in cellular changes, than ions present in the extracts. The osteocalcin staining results are in line with the gene expression study (Fig. 3F), that suggested that *OSTEOCALCIN* expression was not regulated by the extracts, as similarly seen with B25 and B50 borosilicate glasses [24].

It was observed that the cultures exposed to strontium-containing medium started to present slight mineralization after 14 culturing days. These results can be correlated to the role of strontium ions in promoting mineralization in human osteoblast cultures [20, 51]. After 21 culturing days, some mineralization was observed also on other borosilicate cultures.

## Conclusions

In studies by Ojansivu et al., it has been demonstrated that borosilicate glasses B25 and B50 had favourable effect on hADSC commitment towards osteogenic cell line, though having strong inhibitory effect on cell proliferation. In the current study, experiments were continued with a borosilicate glass series B12.5, that contained less boron (substituted for SiO_2_) with additional Mg/Sr to Ca substitution in their composition. While the glass degradation products changed the ion concentrations of the culture media, they additionally introduced new elements to the cells; boron and silicon from all of the borosilicate extracts, and in some conditions, additionally strontium. Overall, the degradation products were highly tolerated; all cultures stayed viable and reached high cell densities. While borosilicate B12.5 ion extracts resulted in slower proliferation than on silicate S53P4 cultures, Mg/Sr for Ca substitution in the B12.5 composition slightly increased the proliferation, and especially Sr for Ca (without added Mg substitution) yielded highest cell numbers in the study. Additionally, viability assay showed that cells stayed highly viable when cultured in direct contact with the borosilicate glasses.

Even though osteogenic media supplements were utilized to guide the differentiation towards osteogenic lineages, the ion extracts had a notable effect on the cell’s behaviour; for example, the cells ALP activity and several osteogenic marker genes were upregulated by the degradation products. The gene expression studies and mineralization assay indicated, that while the cultures did not reach major ECM mineralization upon 21 days of culturing, yet the degradation products had favourable effects on hADSC differentiation process and ECM maturation. Additionally, when hADSCs were cultured on top of glass discs, while the OM supplements did facilitate the cells collagen-I production, the collagen-I production in BM cultures was however on similar level as observed with OM supplemented extracts cultures. This indicated that the direct contact with the bioactive glasses was alone enough to induce the hADSC differentiation, emphasising the role of glass surface physico-chemical properties in promoting osteogenesis.

Bioactive glasses, especially boron containing ones, are known to support angiogenesis. Indeed, also the B12.5-series had a notably upregulating effect in the endothelial marker *vWF* and *PECAM-1* expression. Moreover, as the especially *PECAM-1* upregulation was more pronounced with higher Mg/Sr for Ca substitution in the glass composition while the level of B was similar in all extracts, the presence of Mg and/or Sr or the ratio between Sr/B, Ca/B and/or Mg/B might play an important role on the upregulation of angiogenic factors.

In the studied borosilicate glasses, part of the calcium has been gradually substituted with varying amounts of magnesium and strontium. As the ion concentrations of the produced extracts varied based on glass composition, it is challenging to attribute clear trends specifically to a particular composition/property relationship. It however could be noted that for this specific glass series, the magnesium and strontium for calcium substitution in the composition had a slightly more prominent effect on cells, than the pure borosilicate. As a conclusion, it was observed that all glasses and their degradation products had a beneficial effect on hASDCs osteogenic commitment, while simultaneously supporting angiogenic factors. Thus, B12.5 glass series could be considered favourable for bone tissue engineering applications.
